# Supplementary figures and images for: Mitochondrial ATP production promotes T cell differentiation and function by regulating chromatin accessibility
Source: bioRxiv. 2026 Mar 28:2026.03.27.714789. Preprint. [Version 1] doi: 10.64898/2026.03.27.714789 (PMC13041975; doi:10.64898/2026.03.27.714789)

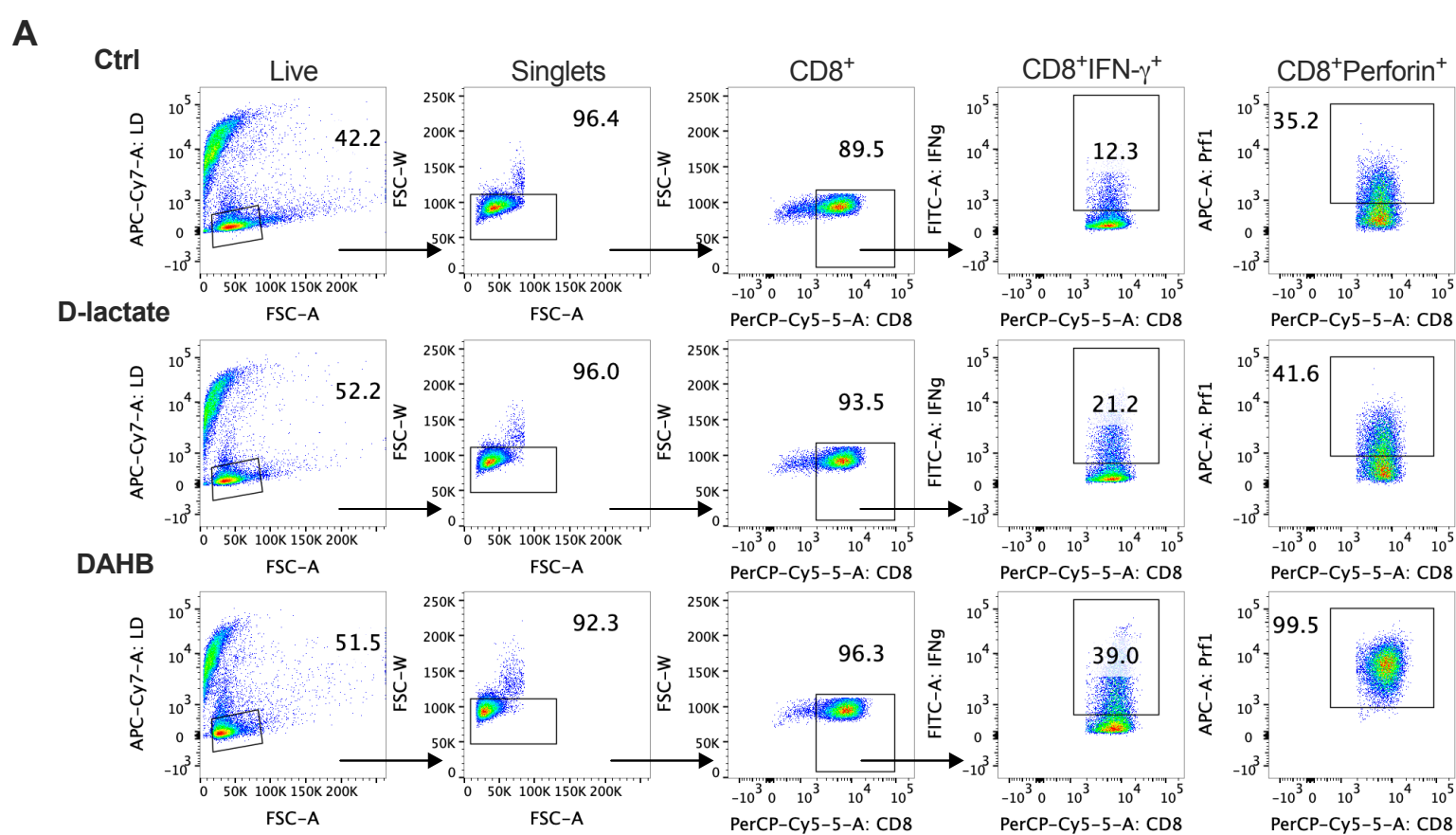

**B**

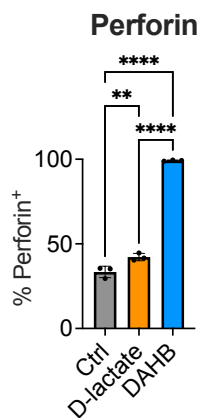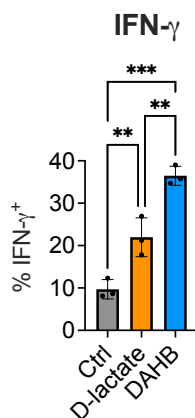

**C**

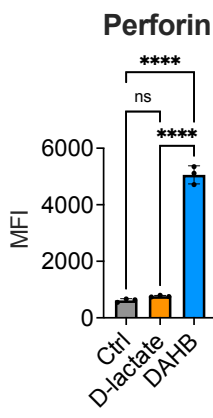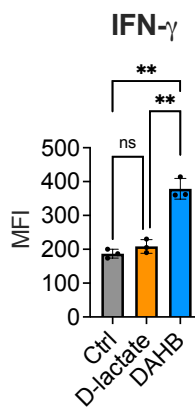

**D**

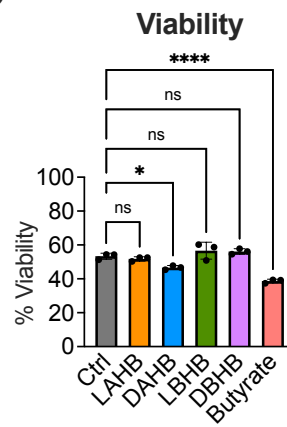

**E**

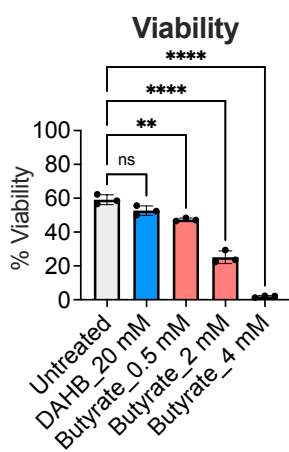

**F**

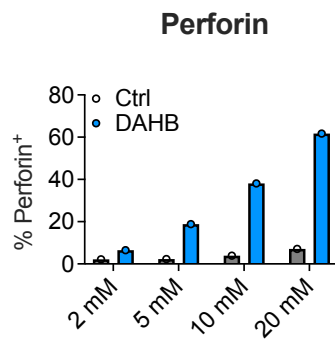

**G**

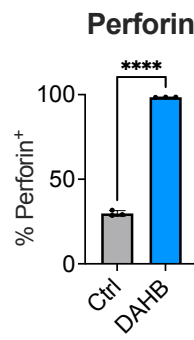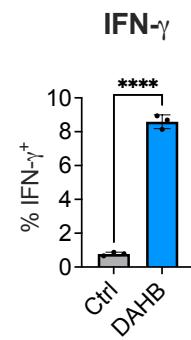

**H**

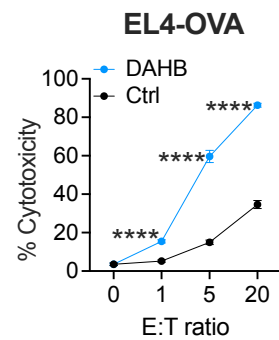

Figure S1

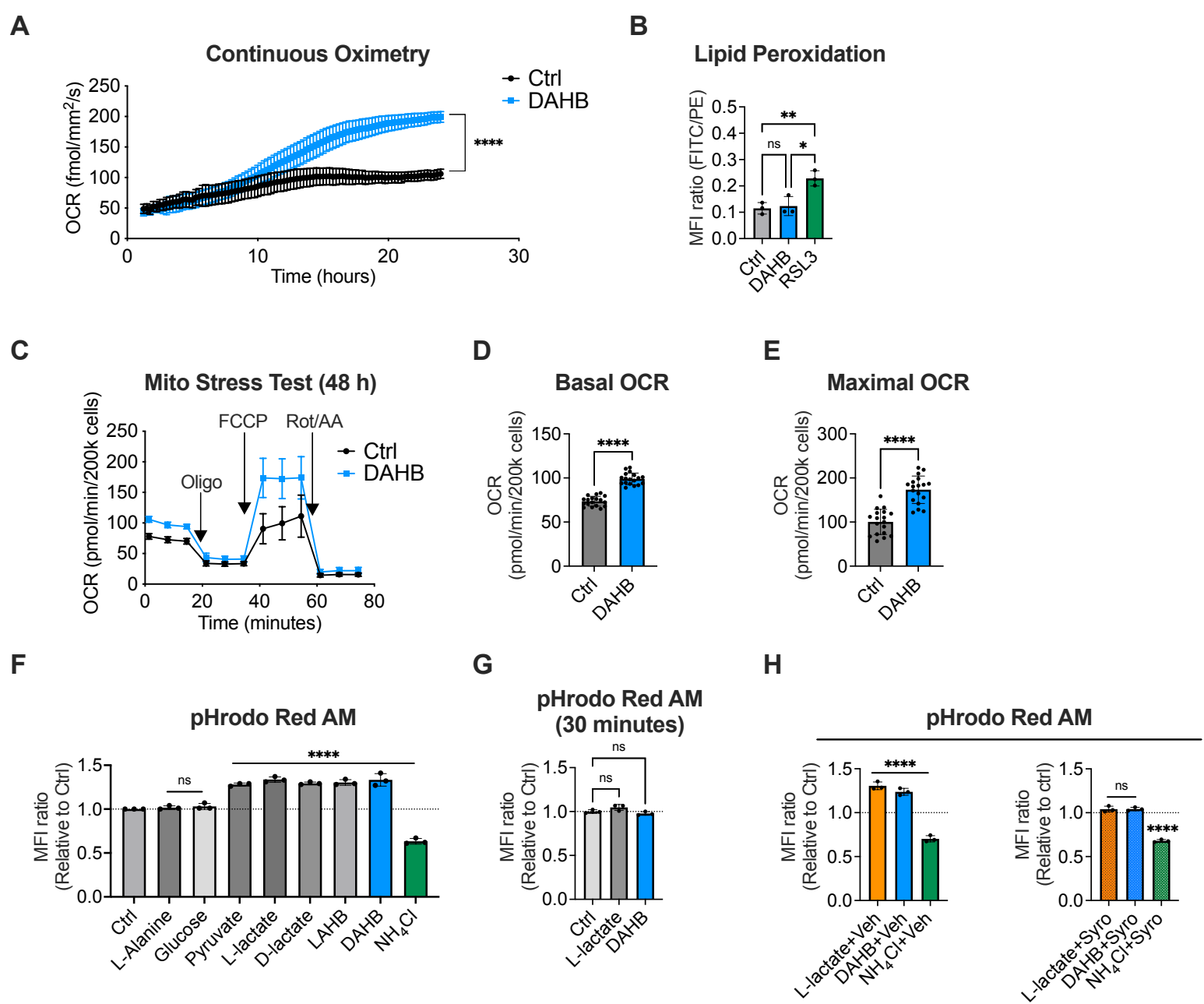

Figure S2

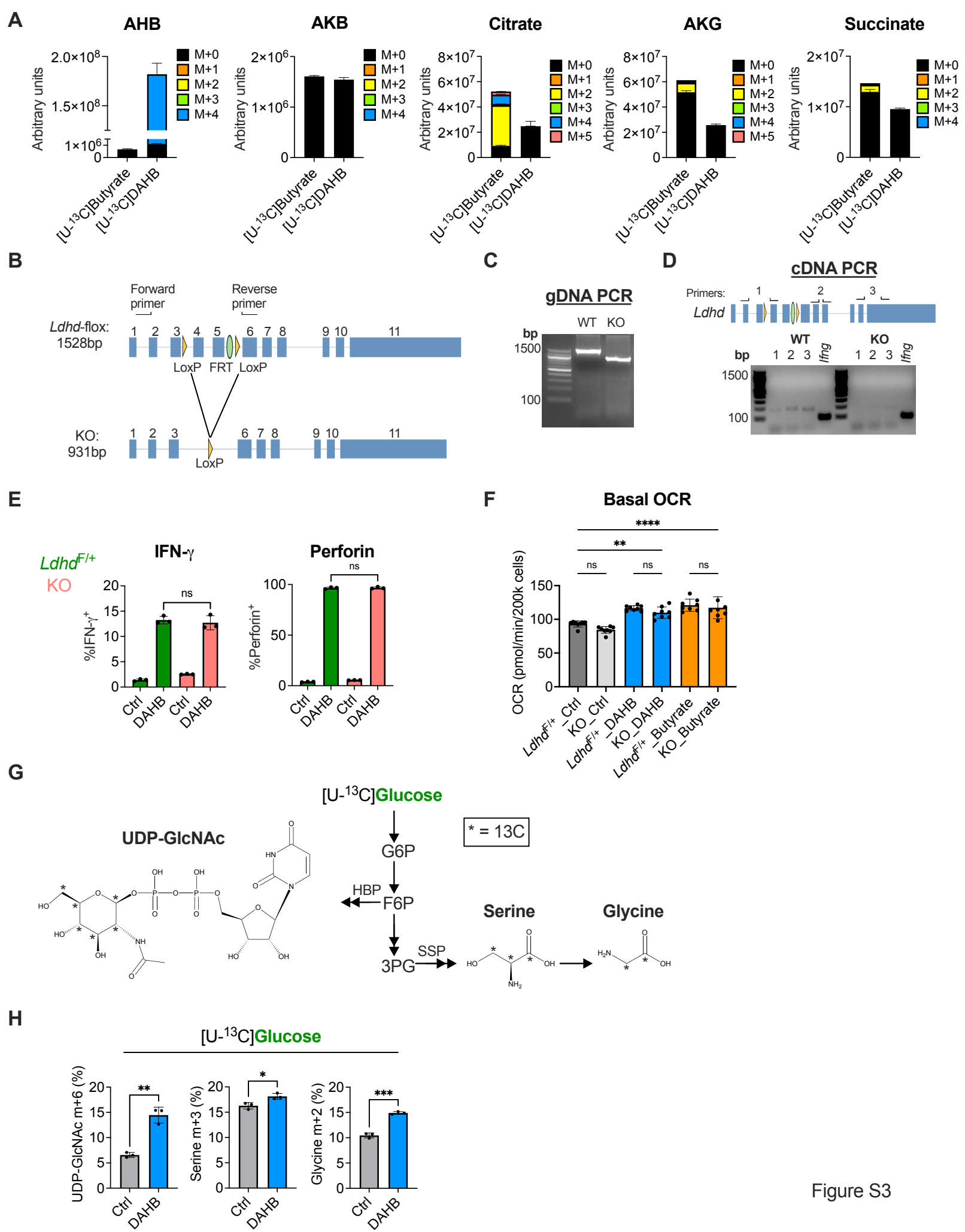

Figure S3

**A**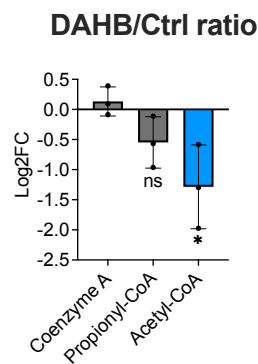**B**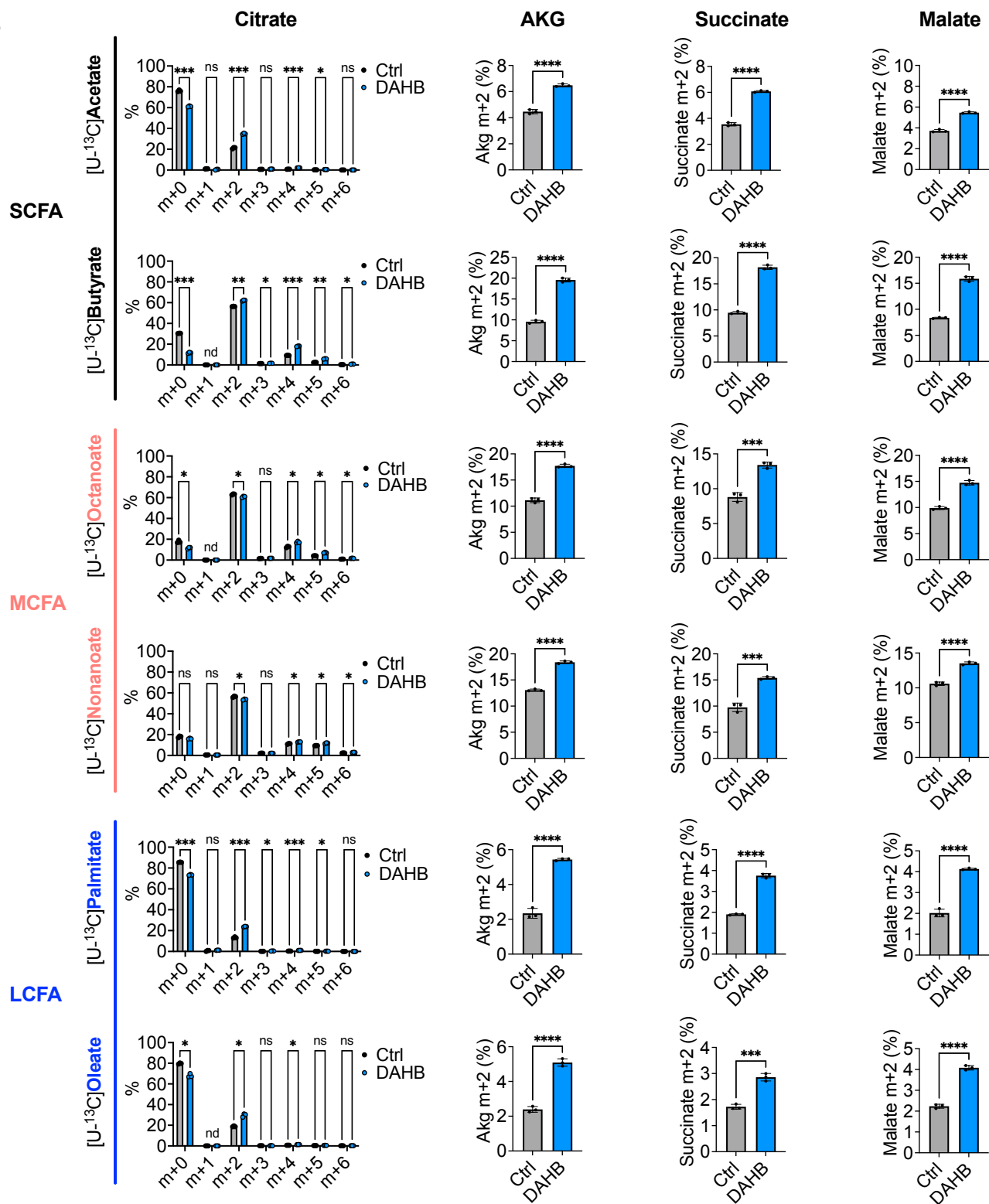

Figure S4

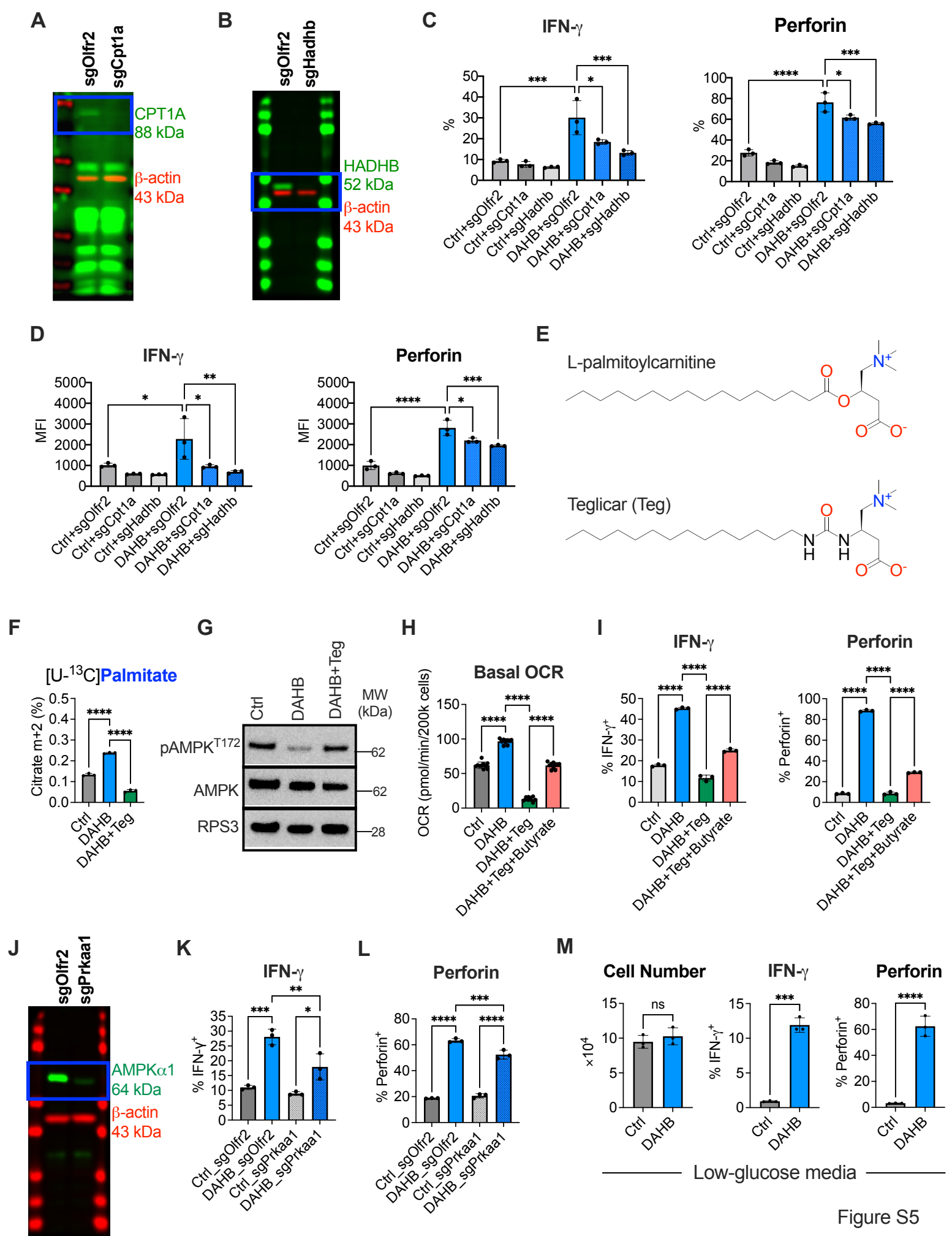

Figure S5

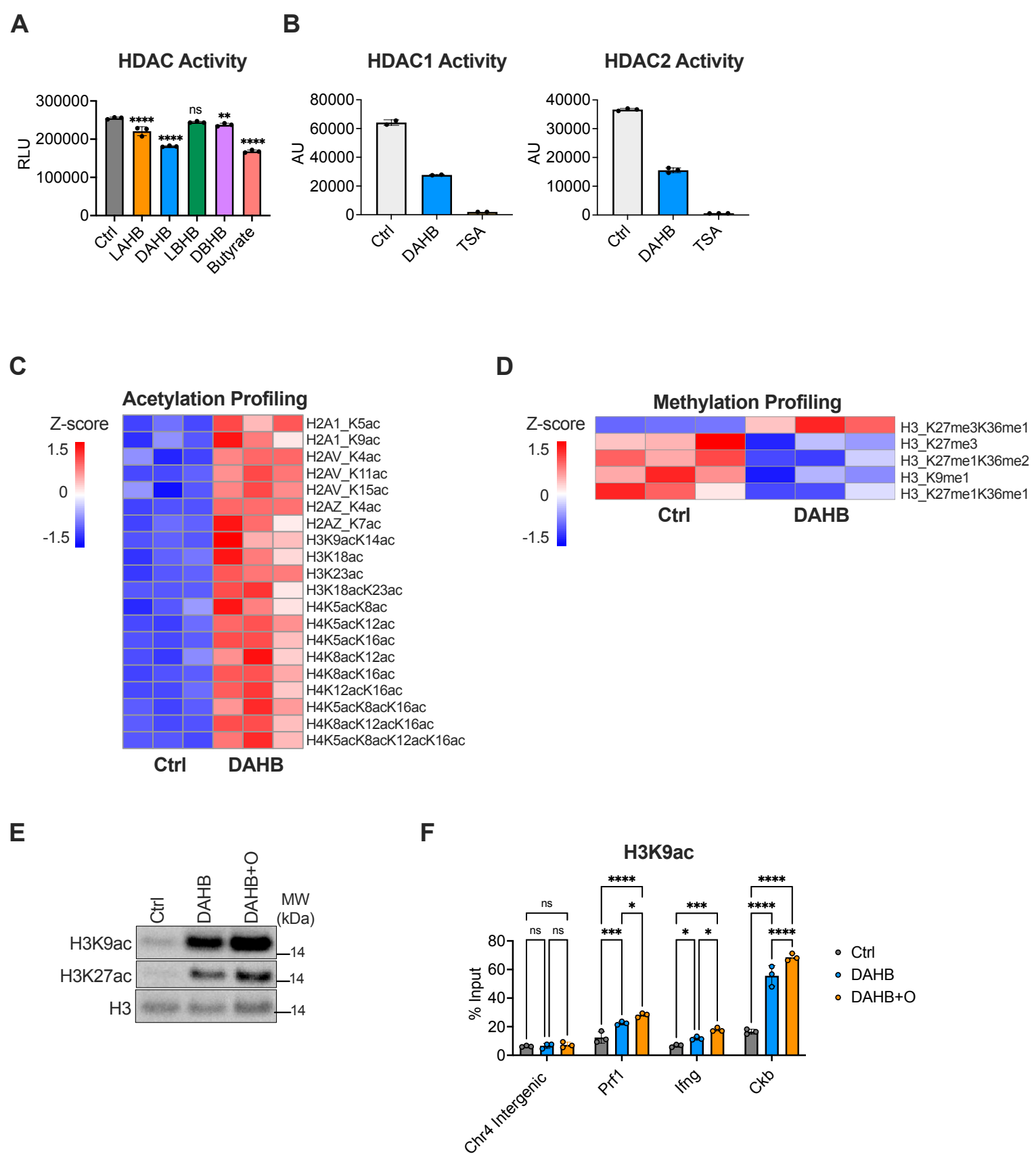

Figure S6

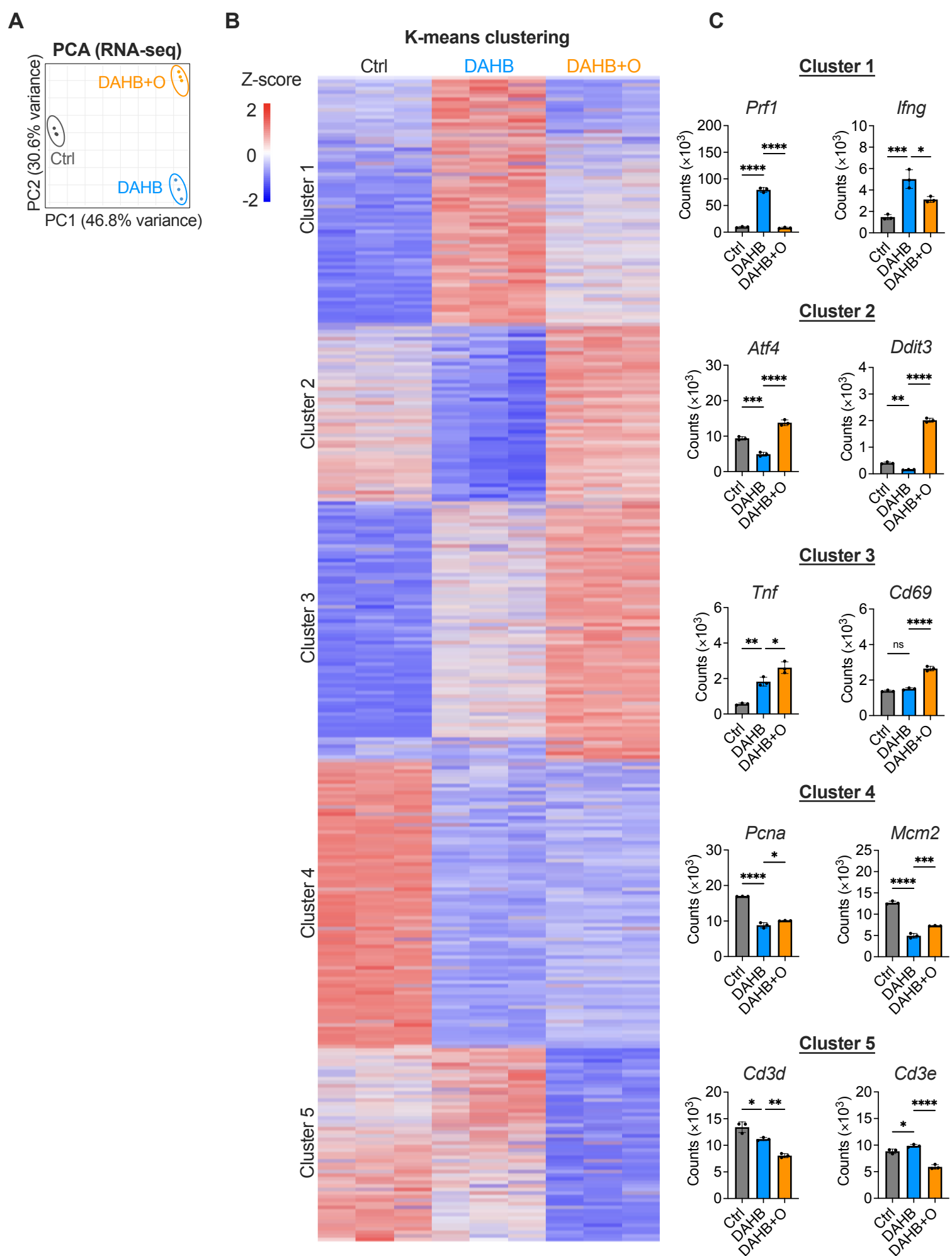

Figure S7

**A**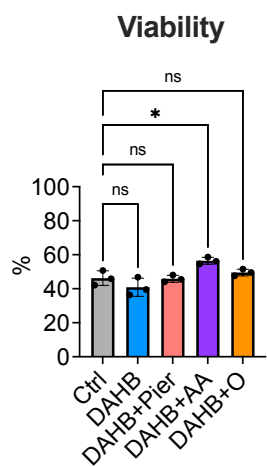**B**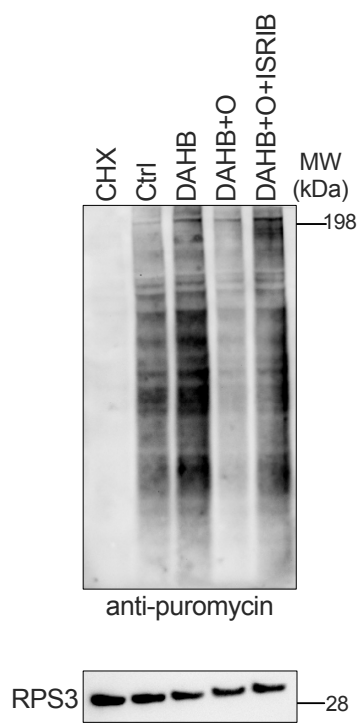



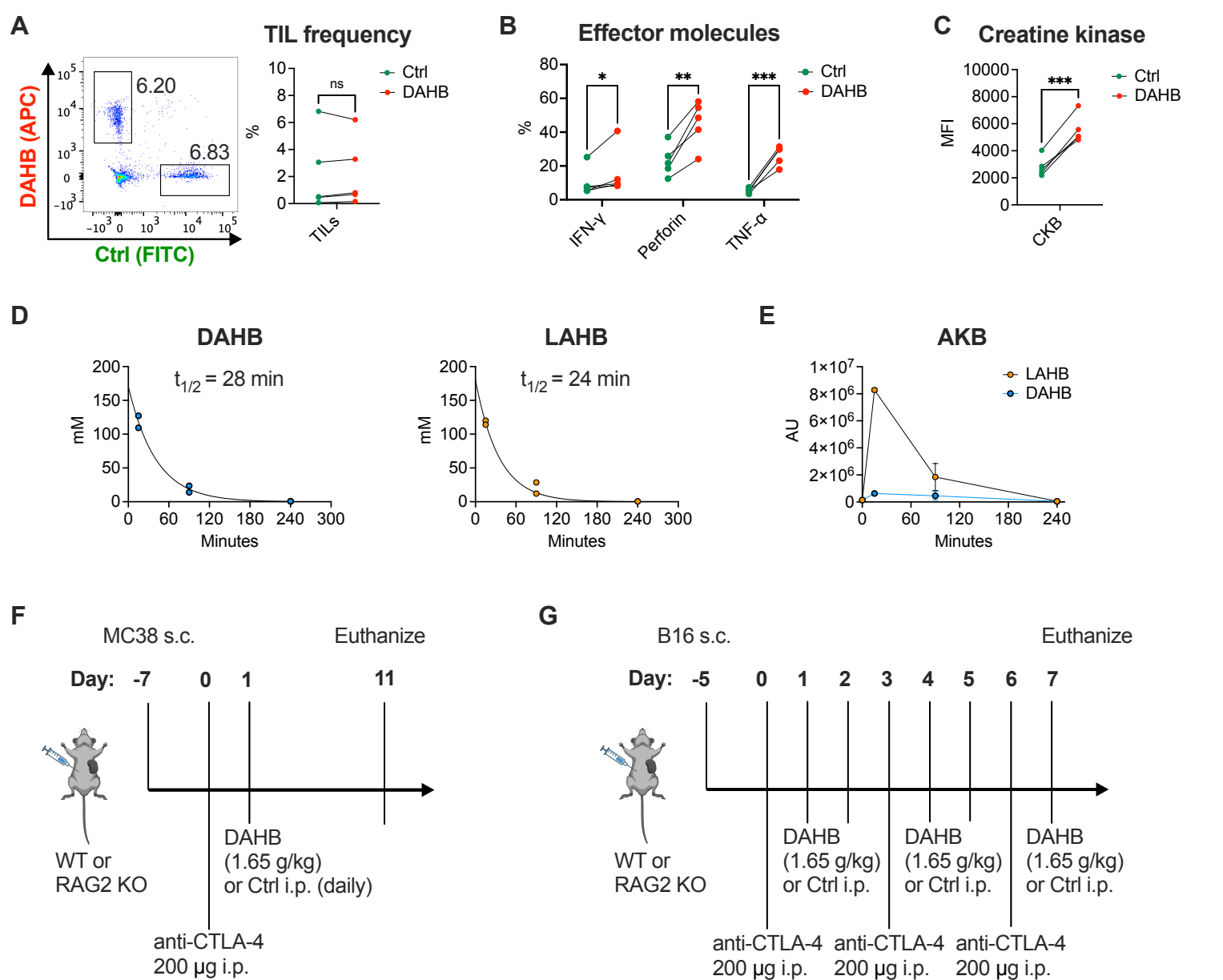

Figure S10

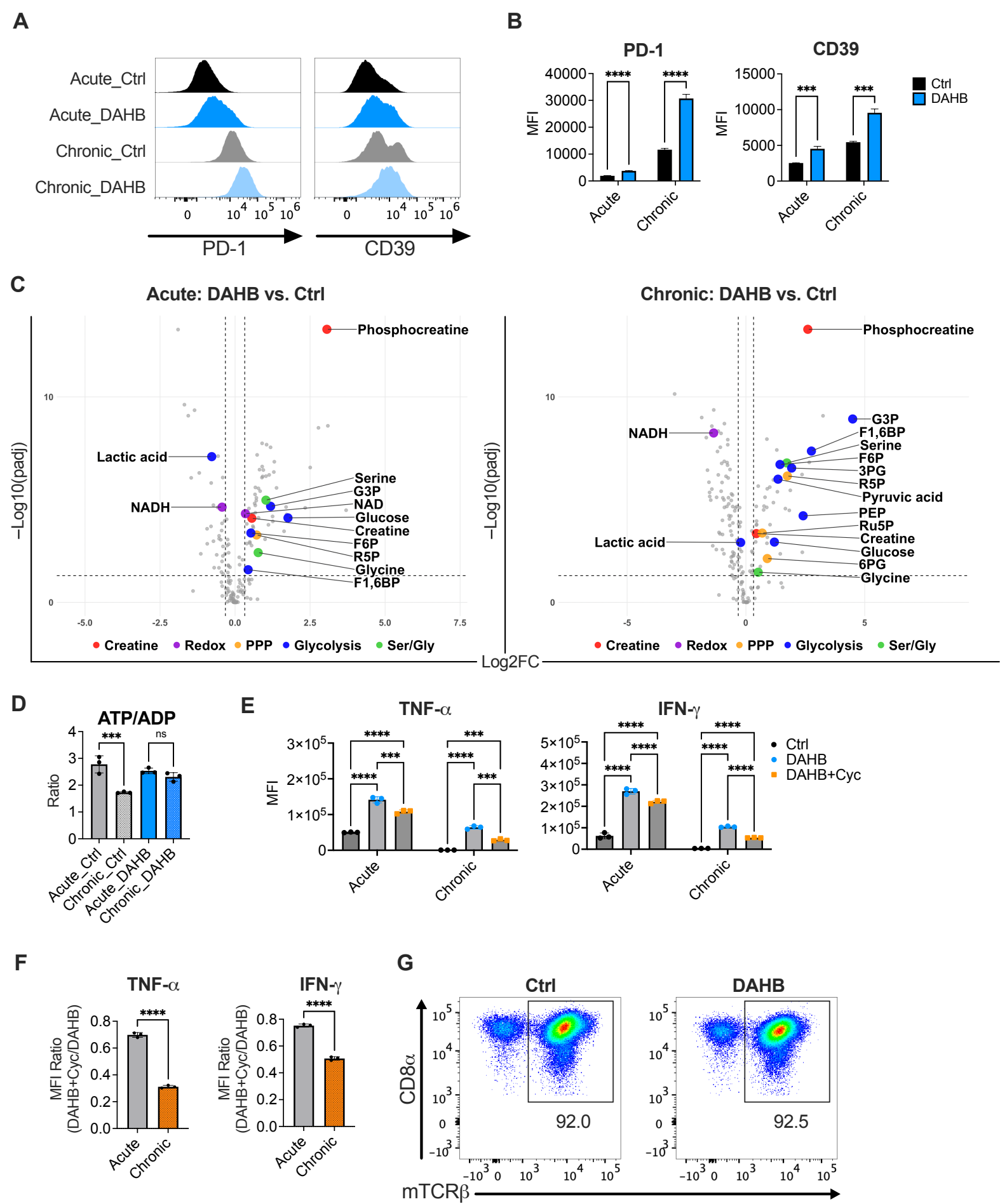

Figure S11

Supplement: Supplement 1 — Figure S1. DAHB enhances durable effector function without significant toxicity, related to Figure 1. (A) Representative gating strategy to determine cytokine production in activated CD8+ T cells treated with Ctrl, D-lactate or DAHB. (B and C) Perforin and IFN-γ production by percent positive (B) or MFI (C) of CD8+ T cells treated as in Figure 1B (n = 3). (D) Viability using live/dead dye after indicated treatments (20 mM for LAHB, DAHB, LBHB, DBHB and 0.5 mM for butyrate); n = 3. (E) Viability using live/dead dye during butyrate titration (0–4 mM); n = 3. (F) Perforin production by flow cytometry after DAHB titration (2–20 mM) compared with Ctrl. Data are representative of three independent experiments. (G) Activated CD8+ T cells were treated for 24 h with Ctrl or DAHB, washed and cultured for an additional 24 h prior to restimulation and measurement of Perforin and IFN-γ by flow cytometry (n = 3). (H) OT-I CD8+ T cells pretreated with Ctrl or DAHB and assessed for cytotoxicity against EL4-OVA via titration of effector-to-target (E:T) cell ratios (0:1, 1:1, 5:1, 20:1); n = 3. Data are mean ± SD. Statistical analysis was performed by one-way ANOVA with Tukey’s multiple comparisons test (B–E) or unpaired two-tailed Student’s t test (G,H; calculated from each E:T ratio for H). ns, not significant; *p < 0.05; **p < 0.01; ***p < 0.001; ****p < 0.0001. Figure S2. DAHB entry is mediated by monocarboxylate transporters (MCTs) and induces prolonged effects on OXPHOS, related to Figure 2. (A) Oxygen consumption measured by continuous oximetry over 24 h in activated CD8+ T cells with treatment of Ctrl or DAHB initiated at time 0 (n = 8 wells per condition). (B) Lipid peroxidation after 24 h treatment with Ctrl or DAHB in activated CD8+ T cells as measured by the FITC/PE MFI ratio of BODIPY C11 staining; n = 3. RSL3, an inducer of lipid peroxidation, was added to untreated wells prior to measurement. (C) Seahorse plot of oxygen consumption rate (OCR) in activated CD8+ T cells [file media-1.pdf]
